# Zoonotic Sporotrichosis in Paraguay: A Public Health Alert

**DOI:** 10.1111/myc.70130

**Published:** 2025-12-17

**Authors:** Mirtha Gabriela Santacruz Silvero, Carolina Melchior do Prado, Bram Spruijtenburg, Amiliana Pineda, Olga Aldama, Azucena Lezcano, Maria Leticia Ojeda, Nancy Segovia Coronel, Caroline Amaral Martins, Federico Augusto Lacarrubba Codas, Liz Scheid, José María Duarte Zacarías, Derlis Rojas, Arnaldo Aldama, Ana Buongermini Gotz, Vania Aparecida Vicente, Jacques F. Meis, Theun de Groot, Flávio Queiroz‐Telles, Eelco F. J. Meijer, José Pereira Brunelli

**Affiliations:** ^1^ Regional Epidemiological Laboratory, Faculty of Health Sciences National University of the East Minga Guazú Paraguay; ^2^ Postgraduate Program in Microbiology, Parasitology and Pathology, Biological Sciences, Department of Basic Pathology Federal University of Parana Curitiba Brazil; ^3^ Radboudumc‐CWZ Center of Expertise for Mycology Nijmegen the Netherlands; ^4^ Department of Medical Microbiology and Immunology Canisius‐Wilhelmina Hospital (CWZ)/Dicoon Nijmegen the Netherlands; ^5^ Department of Medical Microbiology Radboudumc Nijmegen the Netherlands; ^6^ Dermatological Specialties Center Ministry of Public Health and Social Welfare San Lorenzo Paraguay; ^7^ Zoonosis Control Center Foz do Iguaçu Brazil; ^8^ Department of Infectology Tesãi Foundation Ciudad del Este Paraguay; ^9^ Department of Dermatology Tesãi Foundation Ciudad del Este Paraguay; ^10^ Regional Hospital of Ciudad del Este, X Health Region Ministry of Public Health and Social Welfare Ciudad del Este Paraguay; ^11^ Dermatology Service Itauguá National Hospital Itauguá Paraguay; ^12^ Department of Dermatology, Faculty of Medical Sciences National University of Asunción San Lorenzo Paraguay; ^13^ Institute of Translational Research, Cologne Excellence Cluster on Cellular Stress Responses in Aging‐Associated Diseases (CECAD), Excellence Center for Medical Mycology (ECMM) University of Cologne Cologne Germany; ^14^ Department of Public Health, Hospital de Clínicas Federal University of Paraná Curitiba Brazil

**Keywords:** ant‐associated sporotrichosis, emerging disease, genotyping, *S. brasiliensis*, spread, zoonosis

## Abstract

**Background:**

Cat‐transmitted sporotrichosis caused by *Sporothrix brasiliensis* is increasingly reported in the last three decades within Brazil. Recently, other South American countries like Argentina and Chile also reported cases, while the number of reported cases in Paraguay, a Brazilian neighbour, remains quite limited with 10 feline and three human cases. Importantly, early diagnosis and disease awareness facilitate effective treatment and allow strategies to prevent spread.

**Objectives:**

Here, we describe two previously reported and 11 novel human sporotrichosis cases by *S. brasiliensis* from Paraguay, diagnosed from 2017 to 2025.

**Methods:**

Clinical and epidemiological data of patients were collected, fungal isolates were phenotypically analysed with microscopy, and short tandem repeat (STR) genotyping was used for species identification and to determine genetic relatedness between isolates.

**Results:**

From the 13 human patients, 11 were diagnosed with sporotrichosis after contact with cats, while two reported ant bites as the source. All patients reported subcutaneous lesions with lymphocutaneous spread and were treated successfully, resulting in complete resolution of the lesions, despite late recognition of the disease and prior antibiotic treatment. STR genotyping revealed a unique genotype for four cases, all imported from Brazil, including the two ant‐associated isolates, all patients had a history of first symptoms while still in Brazil, but later after moving to Paraguay the diagnosis was made. All other isolates were allocated to the previously identified Rio de Janeiro (RJ) clade, originating from Brazil and known to be more widespread in Brazil.

**Conclusions:**

Altogether, we report the probable first two cases of transmission by ants for *S. brasiliensis* , and all cases result from direct import or spread from Brazil.

## 1 Introduction

Sporotrichosis caused by *Sporothrix brasiliensis* is a saprozoonosis and implantation mycosis that in the last 30 years has become a major public health issue in Latin America. There are a significant number of cases in Brazil, the epicentre of this outbreak, and *S. brasiliensis* cases have recently been reported in other countries, such as Argentina, Paraguay, and Chile [1]. Imported cases were also described recently in the United Kingdom [2]. *S. brasiliensis* is considered the most virulent species within the clinically relevant clade of the *Sporothrix* genus, which also includes *S. schenckii* , *S. globosa* and *S. luriei* [3]. Since the 90s outbreak in Rio de Janeiro, *S. brasiliensis* has become the predominant species responsible for zoonotic sporotrichosis in both cats and humans [3, 4].

*S. brasiliensis* is mainly associated with zoonotic transmission (cat‐to‐human) through direct inoculation via scratches, bites, contact with exudate and respiratory droplets of sick cats [5, 6]. Transmission through contact with organic materials (sapronotic route) was also described as probably associated with *S. brasiliensis* infections. Another probable route is through contact with fomites [5]. Although scratches by asymptomatic cats are implied in transmission, further research is needed to assess to which extent colonisation and fungal loads are sufficient for transmission. It has been demonstrated that introductions by *S. brasiliensis* into previously unaffected regions are usually due to the movement of potentially infected or colonised animals. One example is the United Kingdom, where three cases of cat‐transmitted sporotrichosis were documented after contact with a cat imported from Brazil [2]. A similar event occurred in Paraguay, in the Asunción metropolitan area, in 2017 when two members of a family were infected following the relocation of their pet from Brazil [7].

In Paraguay, the first cases of sporotrichosis in felines were reported in 2023 in the eastern part of the country [8]. The following year, the first autochthonous case, defined as having no travel history and only contact with a cat also without a travel history, of zoonotic sporotrichosis in a human patient was reported in the same region [9]. Of note, both reports were from the border region with Brazil and Argentina, the latter also reporting numerous cases, indicating spread across borders given that the countries are connected by bridges across the major river [10]. Because sporotrichosis is not mandatory to report to central healthcare authorities, the current epidemiological situation in Paraguay remains largely unknown. This study aimed to investigate the introduction and spread of zoonotic sporotrichosis by analysing the demographics, epidemiology and genetic relatedness of isolates from 13 human cases in Paraguay, of which the first two cases were previously reported without providing clinical details and genotyping results [7].

## 2 Materials and Methods

### 2.1 Ethics

All clinical and demographic patient data were collected in accordance with the Declaration of Helsinki. Patients signed consent forms to allow the publication of their case information. The protocol was approved by the Ethics Committee of the National University of the East, Faculty of Health Sciences, Minga Guazú, Paraguay (Resolution No. 115/2025—Acta 562).

### 2.2 Case Definition

For this study only confirmed cases were included using the following case definition.

Confirmed case: isolation of *S. brasiliensis* by fungal culture from lesions compatible with sporotrichosis.

### 2.3 Patients and Sample Collection

For diagnosis of their skin infection, all patients attended the laboratory at the Medical Research Center in Minga Guazú, Paraguay and the Dermatology Specialties Center in San Lorenzo, Paraguay. Four patients were identified through community outreach campaigns, which provided information on the disease, including its clinical manifestations in humans and animals, prevention, care in the event of contact with infected animals and guidance on where to seek help if symptoms appeared. The other nine patients were referred by specialist professionals.

Patients were submitted to the clinical evaluation, and the following data were analysed: age, sex, city (geographic coordinate), occupation, date of first symptoms, date of diagnosis, type of lesion, clinical form, exposure/form of transmission, treatment regimen and treatment duration.

Lesion samples were obtained using a scalpel, collecting purulent secretions in most cases by expressing the exudate under pressure without incisions. When secretion was absent, crusted lesions were gently scraped or crusts carefully removed to facilitate sampling. In addition, tissue biopsies were performed in selected cases to obtain representative material.

### 2.4 Diagnosis

Samples of purulent secretions and tissue biopsies were plated directly onto Sabouraud dextrose agar (SDA) supplemented with 100 mg/L chloramphenicol and incubated at 25°C ± 2°C for up to 30 days. The macromorphological and micromorphological aspects were observed. Isolates were stored at −70°C according to standard procedures for further analysis.

### 2.5 Short Tandem Repeat (STR) Genotyping

Prior to genotyping, isolates were subcultured on SDA for 7 days at 25°C. Next, DNA was extracted using MightyPrep Reagent for DNA (Takara Bio Inc., Shiga, Japan) as previously outlined [11]. Three multiplex PCRs, each amplifying three microsatellite markers were conducted under conditions described earlier [12]. Amplicons were diluted 1000× and 0.12 μL Orange‐600 DNA Size Standard was added (Nimagen, Nijmegen, the Netherlands). These products were run on a 3500 XL genetic analyser (Applied Biosystems, Foster City, CA, USA) and copy numbers were called using GeneMapper 5 (Applied Biosystems). Phylogenetic relationship was investigated with BioNumerics software v7.6.1 (Applied Maths NV, Sint‐Matems‐Latem, Belgium) [12]. Current isolates were compared with previously genotyped isolates to assess the population structure [13].

## 3 Results

### 3.1 Clinical and Epidemiological Data

A total of 13 patients diagnosed with sporotrichosis between April 2017 and May 2025 in Paraguay, were included in the present study. Between 2017 and 2022, five cases were diagnosed in San Lorenzo (Asunción metropolitan area). The latter patients were living in São Paulo, Brazil, when the first signs of the disease were noted, moving later to Paraguay where the diagnosis was made. Three patients stated having contact with symptomatic Brazilian cats in 2017 (*n* = 2) and 2022 (*n* = 1) and two patients indicated ant bites as the origin of the lesions in 2022. Of these five imported cases, two patients were from the same family and also moved their cat to Paraguay. Two other patients were also from the same family but did not own a cat at the time of disease onset.

**FIGURE 1:**
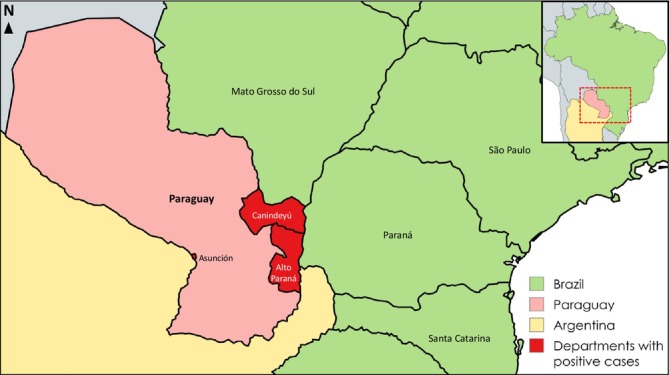
Geographic distribution of the cases.

From 2024 to 2025, there were seven autochthonous cases of sporotrichosis from the metropolitan area of Ciudad del Este, including the cities of Presidente Franco in Alto Paraná Department, a region that shares borders with the Brazilian state of Paraná, which were all cat transmitted. Additionally, in 2025, an autochthonous cat‐associated case from Salto del Guairá in the Canindeyú Department, which borders the Brazilian states Mato Grosso do Sul and Paraná, was reported (Figure 1). The eight autochthonous cases acquired the infections in their own regions without travel history outside of Paraguay and also had only contact with Paraguayan cats without travel history and with symptoms of sporotrichosis. Patients had diverse occupations, with two working in a veterinary setting. The occupations of other patients were not related to animals or plants. Of the 13 patients, two were paediatric (< 18 years) and the remaining ones adults (varying from 9 to 65 years) and regarding sex, seven were female.

Lesions were mostly located on the upper limbs (*n* = 9), including the hand and arm; followed by the lower limbs (*n* = 2), such as the foot, ankle, calf and thigh; one patient had arm and leg lesions, and another one abdominal (Table 1). Regarding the clinical manifestation, 10 patients presented with lymphocutaneous disease, while three had a fixed cutaneous form. The time between the first symptoms and the diagnosis varied between 1 and 2 months. Ten patients received itraconazole orally (50–200 mg/day), two received potassium iodide (3–5 mg/day), while one pregnant patient was treated with terbinafine (500 mg/day). Clinical evolution was favourable in most cases, with clinical resolution varying from 4 to 6 months, with no infection recurrence to date (October 2025). One patient discontinued therapy, while three were still undergoing treatment at the time of this study (Table 1).

**TABLE 1:** Clinical data.

Case	Age category^b^	Sex	Work associated with veterinary setting	Time between first symptoms and diagnosis (months)	Year of diagnosis	Type of lesion	Region affected	Clinical form	Zoonotic transmission	Treatment	Treatment duration
1^a^	Adult	M	No	2	2017	Lymphangitic	Arm	Lymphocutaneous	Yes (cat)	PI 5 g/day	5 months
2^a^	Child	M	NA	1	2017	Erythematous	Abdomen	Fixed cutaneous	Yes (cat)	PI 3 g/day	5 months
3^a^	Adult	M	No	2	2022	Lymphangitic	Leg	Lymphocutaneous	Yes (ant bites)	Itra 200 mg/day	4 months
4^a^	Adult	F	No	2	2022	Erythematous	Both arms	Lymphocutaneous	Yes (ant bites)	Itra 200 mg/day	4 months
5^a^	Adult	M	No	1	2022	Lymphangitic	Arm	Lymphocutaneous	Yes (cat)	Itra 200 mg/day	5 months
6	Adult	F	No	1	2024	Lymphangitic	Arm + leg	Lymphocutaneous	Yes (cat)	Itra 200 mg/day	6 months
7	Adult	M	Yes	1	2024	Lymphangitic	Arm	Lymphocutaneous	Yes (cat)	Itra 200 mg/day	6 months
8	Adult	F	Yes	15 days	2024	Single	Arm	Fixed cutaneous	Yes (cat)	Itra 200 mg/day	4 months
9	Adult	M	No	4	2024	Lymphangitic	Arm	Lymphocutaneous	Yes (cat)	Itra 200 mg/day	4 months
10	Adult	F	No	2	2025	Single	Leg	Fixed cutaneous	Yes (cat)	Terb 500 mg/day	15 days^c^
11	Adult	F	No	1	2025	Lymphangitic	Hand + arm	Lymphocutaneous	Yes (cat)	Itra 200 mg/day	Ongoing
12	Adult	F	No	2	2025	Lymphangitic	Arm	Lymphocutaneous	Yes (cat)	Itra 200 mg/day	Ongoing
13	Child	F	NA	2	2025	Lymphangitic	Both arms	Lymphocutaneous	Yes (cat)	Itra 50 mg/day	Ongoing

Abbreviations: F, female; Itra, itraconazole; M, male; PI, potassium iodide; Terb, terbinafine.

^a^ Imported cases from Brazil.

^b^ Child (< 18 years), adult (≥ 18 years).

^c^ Treatment interrupted.

### 3.2 Microscopic Analysis

Pure cultures were obtained in all cases except those involving scraped samples, where commensal skin flora were also present. Colonies varied from membranous to velvety with short aerial mycelium. The colony colour transitioned from white to dark brown over time. Fungal growth became evident around Day 5, with full development between Days 7 and 10. Septate hyaline hyphae were observed, along with oval or pyriform conidia arranged sympodially at the tips of conidiophores, forming the characteristic ‘rose‐petal’ or ‘daisy‐flower’ clusters, confirming the identification of the agent as *Sporothrix* spp.

### 3.3 Species Identification and Genetic Relatedness

**FIGURE 2:**
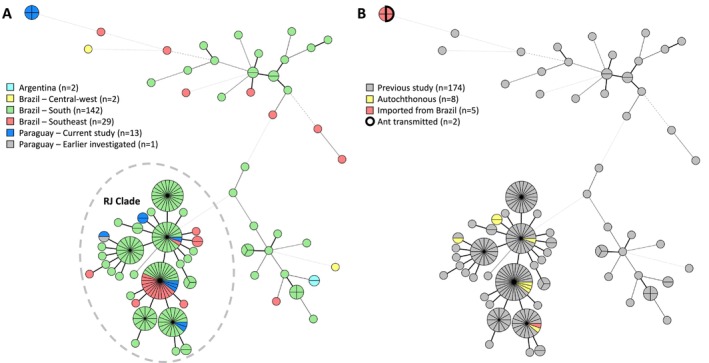
Minimum‐spanning tree of 189 *Sporothrix brasiliensis* isolates, including the 13 cases from Paraguay in this study. Isolates were coloured after their geographic origin (A) or introduction route (B). Branch lengths indicate similarity, with thick solid lines (variation in one marker), thin solid lines (variation in two markers), thin dashed lines (variation in three markers) and thin dotted lines (variation in four or more markers). RJ, Rio de Janeiro.

Species identification and phylogenetic relatedness between all 13 current isolates and 183 previously genotyped *S. brasiliensis* isolates were assessed with a *S. brasiliensis* ‐specific STR assay. All 13 isolates were successfully genotyped, identifying *S. brasiliensis* as the causative species. A majority of nine isolates was closely related to each other and differed in two markers at most (Figure 2A). All these isolates and the previously genotyped Paraguayan isolate were allocated to the Rio de Janeiro (RJ) clade, mainly found in South and Southeastern Brazil. Notably, four isolates (cases 1–4) displayed an identical genotype but were not related to any previously found genotype, differing in four or more alleles. Two of these isolates were cat associated from 2017 while the remaining two were ant associated from 2022 (Figure 2B).

## 4 Discussion

In this study, we describe a case series of 11 new and 2 previously reported patients with sporotrichosis caused by *S. brasiliensis* in Paraguay. The five oldest cases in time (2017–2022) were imported while eight more recent cases were all autochthonous, demonstrating the fungus is established within the region and spreading. All five imported cases originated from the State of São Paulo, Brazil, including the first two (from 2017), which were previously reported [7], while the remaining autochthonous eight cases (2024–2025) originated from Brazilian border regions. It is important to highlight that Paraguay is separated from Brazil by the Paraná River and connected by bridges, which allows potential free movement of animals such as cats between the countries. All autochthonous isolates exhibited a RJ genotype, demonstrating that these strains did not emerge from the environment in Paraguay, but probably originated from Brazil, likely via movement of colonised cats. This highlights the need for adequate border policies to screen infected animals, thereby preventing or reducing the introductions into non‐endemic countries like Paraguay [14]. Although most cases are confined to border regions with Brazil, high‐density areas with many felines like the Central Department and its metropolitan area are at risk for new cases, as *S. brasiliensis* is known to affect mostly socially vulnerable populations with a large feline population [15]. Additionally, underdiagnoses due to limited diagnostic capabilities and limited sporotrichosis awareness might underestimate the currently reported cases, particularly in border regions with Brazil in northern Paraguay. All patients were accurately diagnosed from 1 to 4 months after symptom onset, sometimes after failed treatment with empiric antibiotics. Previous reports highlighted the need for rapid diagnosis since untreated cases worsen the clinical presentation [9].

All patients displayed either the lymphocutaneous or fixed cutaneous forms, also most often reported in other countries [16]. Full clinical cure was observed with different agents, with treatment duration ranging from 4 to 6 months. As the observed clinical manifestations were relatively mild, treatment duration was relatively short. In more severe cases, like disseminated ones, treatment may take up to 12 months [13]. Complete clinical cure also indicates absence of antifungal resistance, while reduced antifungal susceptibility is increasingly reported [4]. Interestingly, while most patients did report contact with infected cats, two patients only reported ant bites as the possible source. Corroborating this finding, two reports indicated cases potentially associated with insect bites [17, 18]. To date, only one ant‐associated sporotrichosis case has been reported in the literature [19]. Although this report from the USA did not perform species identification, the cutaneous lesions were successfully cured with itraconazole. Given the species geographic epidemiology, this case was likely caused by *S. schenckii* , which would make the present *S. brasiliensis* cases the first potentially ant associated. The identification of two cases potentially linked to ant bites raises important epidemiological considerations, because they could represent an additional route of transmission, although further investigation is still needed and at present its occurrence is rare. Notably, other *Sporothrix* species have been associated with insects, which reinforces the need for investigation into this option [20].

The two ant‐associated cases from 2022 displayed the same STR genotype as two cases from 5 years earlier. This suggests that in São Paulo, Brazil this genotype is established in the environment, subsequently infecting or colonising ants, albeit to a limited geographic extent, as none of the previously genotyped isolates displayed this genotype. More common was the RJ clade genotype, which is also found throughout Brazil and is responsible for the vast majority of cases in the South and Southeast of the country [12]. Previously, it was hypothesised that this clade harbours an elevated transmission potential when compared to other genotypes [12]. This highlights the need for national surveillance to monitor and halt the spread of this specific genotype.

To prevent and control sporotrichosis in Paraguay increased awareness among health professionals about the disease in humans and animals is required to facilitate early diagnosis and treatment. Especially for cats, early diagnosis would slow down the spread of the disease given that they are the most important source and affected host. Stricter border policies by screening cats that enter the country would prevent introductions to new regions inside Paraguay and control in the areas with reported cases.

To conclude, we report a case series of human sporotrichosis cases, caused by *S. brasiliensis* in Paraguay. Clinical manifestations included lymphocutaneous and fixed cutaneous forms and were successfully treated although the time to diagnosis could be improved. Cases were mainly restricted to border areas with Brazil and often cat associated, indicating that the disease is already established and is currently spreading throughout the country. Moreover, two non‐autochthonous patients reported ant bites as the source of infection, which would be a potentially new zoonotic transmission route for *S. brasiliensis* .

## Author Contributions

**Mirtha Gabriela Santacruz Silvero:** investigation, methodology, visualization, formal analysis, data curation, writing – original draft, writing – review and editing. **Carolina Melchior do Prado:** investigation, writing – original draft, methodology, visualization, writing – review and editing, formal analysis, data curation. **Bram Spruijtenburg:** investigation, writing – original draft, writing – review and editing. **Amiliana Pineda:** investigation, writing – review and editing. **Olga Aldama:** investigation, writing – review and editing. **Azucena Lezcano:** investigation, writing – review and editing. **Maria Leticia Ojeda:** investigation, writing – review and editing. **Nancy Segovia Coronel:** investigation, writing – review and editing. **Caroline Amaral Martins:** writing – review and editing, visualization. **Federico Augusto Lacarrubba Codas:** investigation, writing – review and editing. **Liz Scheid:** investigation, writing – review and editing. **José María Duarte Zacarías:** investigation, writing – review and editing. **Derlis Rojas:** investigation, writing – review and editing. **Arnaldo Aldama:** investigation, writing – review and editing. **Ana Buongermini Gotz:** investigation, writing – review and editing. **Vania Aparecida Vicente:** writing – review and editing. **Jacques F. Meis:** writing – review and editing. **Theun de Groot:** writing – original draft, writing – review and editing, formal analysis, supervision. **Flávio Queiroz‐Telles:** writing – review and editing, supervision, funding acquisition. **Eelco F. J. Meijer:** funding acquisition, writing – original draft, writing – review and editing, formal analysis, supervision. **José Pereira Brunelli:** investigation, writing – original draft, writing – review and editing, methodology, project administration, supervision, formal analysis.

## Conflicts of Interest

E.F.J.M. received research grants from Mundipharma, is on the scientific advisory board for Pfizer and has received speaker fees from Gilead Sciences and Pfizer. All other authors declare no conflicts of interest.

## Data Availability

Data sharing is not applicable to this article as no datasets were generated or analysed during the current study.
